# Pinching or stinging? Comparing prey capture among scorpions with contrasting morphologies

**DOI:** 10.1590/1678-9199-JVATITD-2021-0037

**Published:** 2022-04-01

**Authors:** Luis Fernando García, Juan Carlos Valenzuela-Rojas, Julio César González-Gómez, Mariángeles Lacava, Arie van der Meijden

**Affiliations:** 1 Eastern Regional University Center (CURE), University of the Republic, Treinta y Tres, Uruguay.; 2 Semillero de Investigación INVUSCO, Grupo GIPB, Licenciatura en Ciencias Naturales y Educación Ambiental, Universidad Surcolombiana, Neiva, Huila, Colombia.; 3 Biology and Ecology of Arthropods (BEA) Research Group, Corporación Huiltur, Neiva, Huila, Colombia and Merenberg Foundation, La Plata, Huila, Colombia.; 4 Graduate Program in Biological Sciences, School of Sciences, University of Tolima, Altos de Santa Helena, Ibagué, Tolima, Colombia.; 5 Rivera University Center (CENUR Noreste), University of the Republic (Udelar), Rivera, Uruguay.; 6 BIOPOLIS Program in Genomics, Biodiversity and Land Planning, CIBIO, Campus de Vairão, Vairão, Portugal.

**Keywords:** Bite force, Scorpions, Venom use, Predatory behavior

## Abstract

**Background:**

Scorpions can use their pincers and/or stingers to subdue and immobilize their prey. A scorpion can thus choose between strategies involving force or venom, or both, depending on what is required to subdue its prey. Scorpions vary greatly in the size and strength of their pincers, and in the efficacy of their venom. Whether this variability is driven by their defensive or prey incapacitation functionis unknown. In this study, we test if scorpion species with different pincer morphologies and venom efficacies use these weapons differently during prey subjugation. To that end, we observed *Opisthacanthus elatus* and *Chactas* sp. with large pincers and *Centruroides edwardsii* and *Tityus* sp. with slender pincers.

**Methods:**

The scorpion pinch force was measured, and behavioral experiments were performed with hard and soft prey (*Blaptica dubia* and *Acheta domesticus*). Stinger use, sting frequency and immobilization time were measured.

**Results:**

We found that scorpions with large pincers such as *O. elatus* produce more force and use the stinger less, mostly subjugating prey by crushing them with the pincers. In *C. edwardsii* and *Tityus* sp. we found they use their slender and relatively weak pincers for holding the prey, but seem to predominantly use the stinger to subjugate them. On the other hand, *Chactas* sp. uses both strategies although it has a high pinch force.

**Conclusions:**

Our results show that scorpionspecies with massive pincers and high pinch force as *O. elatus* use the stinger less for prey subjugation than scorpionspecies with slenderpincers.

## Background

Morphology, through its influence on performance, can be linked to some key ecological functions including mating and feeding or defensive behavior [1]. The role of morphology in feeding has been widely explored in vertebrates. For example, some studies have shown that mammal and fish feeding ecology has a stronger evolutionary influence on functional morphology [2,3]. There are a small but increasing number of functional studies exploring the relationship between morphology and feeding ecology in invertebrates, mainly in arachnids and insects [4-8]. A few studies have shown that some parameters,including shape and force, play a key role in prey capture in some predators. For example, crabs with larger claws are able to feed upon larger and harder shells, suggesting a specialization in this prey type [9].

Prey body hardness is considered to be an important defensive mechanism and it is present in a wide variety of animals with hard defensive shells such as armadillos, turtles, alligator and fish [10]. In arthropods, this defensive mechanism is present in numerous taxa, such a beetles, isopods and other arthropods [11-14]. For predators that need to grasp or crush their prey with mandibles, prey hardness represents a challenging parameter. For example, shell hardness might limit the capture ability in some fish and crabs [10,15]. Similarly, some spiders that crush their prey are less efficient than spiders that attack soft spots when capturing hard-bodied prey [16]. Prey morphology is known to determine predatory strategy in several groups including scorpions [17]. Since scorpions use both strategies, piercing soft body parts using the stinger and crushing the body with the pincers (chelae), we consider them a good model to evaluate the effect of prey hardness on feeding strategy. We expect these generalist predators may employ alternative prey capture strategies depending on prey morphology.

Scorpions are a successful group of terrestrial arthropods, present in almost all known terrestrial habitats [18]. Although the high success of this group has been attributed to several traits, morphology plays a key role, particularly their chelae and metasoma that are linked to defense and predation [19]. Among the most characteristic structures in scorpions are the chelae and stinger (telson), which are considered the main weapons and affect several ecological functions in scorpions, including prey capture, defense, sensing and mating [20]. In scorpions, the chelae are pincer-like structures, which vary in strength. The strength correlates with morphological parameters of the chela, particularly the width and height of muscular part of the manus [4]. Pinch force is higher in scorpions with robust chela when compared to species with slender structures. Scorpion morphology is highly variable: some species have robust, powerful chelae, while some others have slender chelae [20]. There are also large differences in the morphology and performance of the stinger and its venom, and the shape and size of the tail-like metasoma that carries the stinger. Scorpions with massive chelae use this structure as their main defensive strategy, whereas scorpions with slender chelae use the stinger more frequently, suggesting a possible tradeoff between these structures [21].

Feeding ecology has been studied for some scorpion species. However, most of these studies have focused on field observations [22-24]. Most studies regarding prey capture have evaluated the role of sting use in prey capture. In some species, sting use can change ontogenetically, with juveniles stinging their prey more frequently than adult individuals [25]. Sting use may also depend on prey activity and resistance, and some species use it only against large, potentially dangerous or highly mobile prey. González-Gomez et al. [6] have shown that scorpions with slender chelae are more toxic for insect prey such as *Tenebrio molitor* Linnaeus, 1758 larvae, which might explain why these scorpions use stinger more frequently in prey capture. However, it is unknown if this pattern is present in other scorpion species and whether it varies depending on prey type. Despite the frequent use of venom in prey capture, scorpions are able to subdue their prey using only the chelae [20]. However, Evans et al. [26] extensively discuss how the mobility and size of prey, presence of predators, and environmental factors such as temperature can affect the use and toxicity of scorpion venom.

The aim of this paper is to evaluate the role of chela force on the prey handling behavior in different scorpion species with contrasting chela morphologies. These range from species with slender and weak chelae to species with robust and very strong chelae. In addition, we evaluate if there is a relationship between chela force and stinger use, and if these traits are dependent on prey type. Since scorpions with stronger chelae tend to use them more [5], we hypothesized that scorpions with stronger chelae would have a lower sting use compared to scorpions with slender chelae. If there exists a tradeoff between sting use and chela morphology, we expect a similar predatory efficiency between species with contrasting morphologies against soft prey, but a higher efficiency of species with strong chelae against hard prey. This as species with robust chelae can, in addition to stinging, use crushing as a means to incapacitate the prey. 

## Methods

### Species selection

We selected four scorpion species with contrasting morphologies. As a species with robust chelae, we selected *Opisthacanthus elatus* (Hormuridae) Gervais, 1844 from San José-Santander Valley (06° 26’53.65’’N 73° 8’20.32’’W), this species is often found under rocks where it makes a shallow burrow. We also selected *Chactas* sp. (Chactidae) from Termales los Ángeles, Rivera-Huila (02° 45’ 06.6”N 75° 14’17.0” W), as in this species the females have robust chelae, and the males have slender chelae. Individuals of this specieswere observed at the entrance of their burrows during the night, especially females. As model species with slender chelae, we chose *Tityus* sp. (Buthidae) collected in a forest in the Universidad Surcolombiana campus in Neiva-Huila (2° 56’40.417”N 75° 18’6.952” W) and *Centruroides edwardsii* (Buthidae) Gervais, 1843 in the Desierto de la Tatacoa, Villavieja-Huila (03° 5’31.61”N 75° 8’25.08” W). We collected a total of 76 specimens (Table 1). Both buthid species were observed actively looking for prey during the night. Although information about the trophic ecology of these species is scarce, preliminary observations suggests all selected scorpion species possess a generalist diet. 

**Table 1.** Morphological characters of males and females of the four species of studied scorpions.

Species	Sex	Morphological characters - All sizes are in mm (mean ± SE)			
		Prosoma		Chela	
		Length	Width	Length	Width
*Centruroides edwardsii*	Female (*n* = 9)	8.3 ± 0.21	7 ± 0.23	13.4 ± 0.51	3.6 ± 0.2
	Male (*n* = 11)	8.8 ± 0.26	6.9 ± 0.23	14.9 ± 0.52	4.1 ± 0.15
*Chactas* sp.	Female (*n* = 8)	6.4 ± 0.16	5.4 ± 0.11	11 ± 0.2	3.7 ± 0.2
	Male (*n* = 8)	6.5 ± 0.14	5.4 ± 0.12	13.9 ± 0.49	3 ± 0.15
*Opisthacanthus elatus*	Female (*n* = 12)	11.7 ± 0.27	11.3 ± 0.37	22.6 ± 0.58	9.2 ± 0.34
	Male (*n* = 8)	11.7 ± 0.46	10.9 ± 0.54	21.4 ± 1.33	8.2 ± 0.45
*Tityus* sp.	Female (*n* = 12)	7.1 ± 0.23	6 ± 0.18	12.6 ± 0.47	2.7 ± 0.08
	Male (*n* = 8)	6.8 ± 0.27	5.8 ± 0.23	13.6 ± 0.58	3.5 ± 0.11

Once collected, all individuals were housed individually in plastic boxes (12x9x6cm). Water was provided *ad libitum* to each scorpion species using wet cotton. Photoperiod (12 light:12 dark), temperature (26°C) and humidity (70%) were held constant during the study. Experiments were done at the BEA laboratory, and voucher specimens were deposited in the Colección Zoológica de la Universidad del Tolima (CZUT).

Collected individuals were randomly assigned to bite force measurement or behavioral experiments using the R software [27].

### Bite force measurement

We randomly selected males and females of each scorpion species, namely:

- *O. elatus* -*n =* 20; 8 males and 12 females;
- *Chactas* sp. -*n =* 20; 7 males and 13 females;
- *Tityus* sp. -*n =* 23; 8 males and 15 females;
- *C. edwardsii* -*n =* 19; 11 males and 8 females.

We measured the bite force using a Kistler low-force sensor, type 9203, connected to a one-channel hand-held charge amplifier, type 5995A (see Additional file 1). Force was transmitted to the sensor by custom-built plates [4,6]. To measure the bite force, scorpions were immobilized except for their chelae which were placed on the sensor plates and the scorpions were stimulated to bite them. Bite force of each pedipalp was measured once per day for five days consecutively. We use only the maximum bite forces of each scorpion for the analysis. The measurements were made at a controlled temperature (25±1°C) following the methods described by González-Gómez et al. [6].

Once bite force was measured, it was compared among the different scorpion species using a linear model with the scorpion species and sex as explanatory variables, while log-transformed force was used as response variable. 

### Behavioral experiments

**Figure 1.**
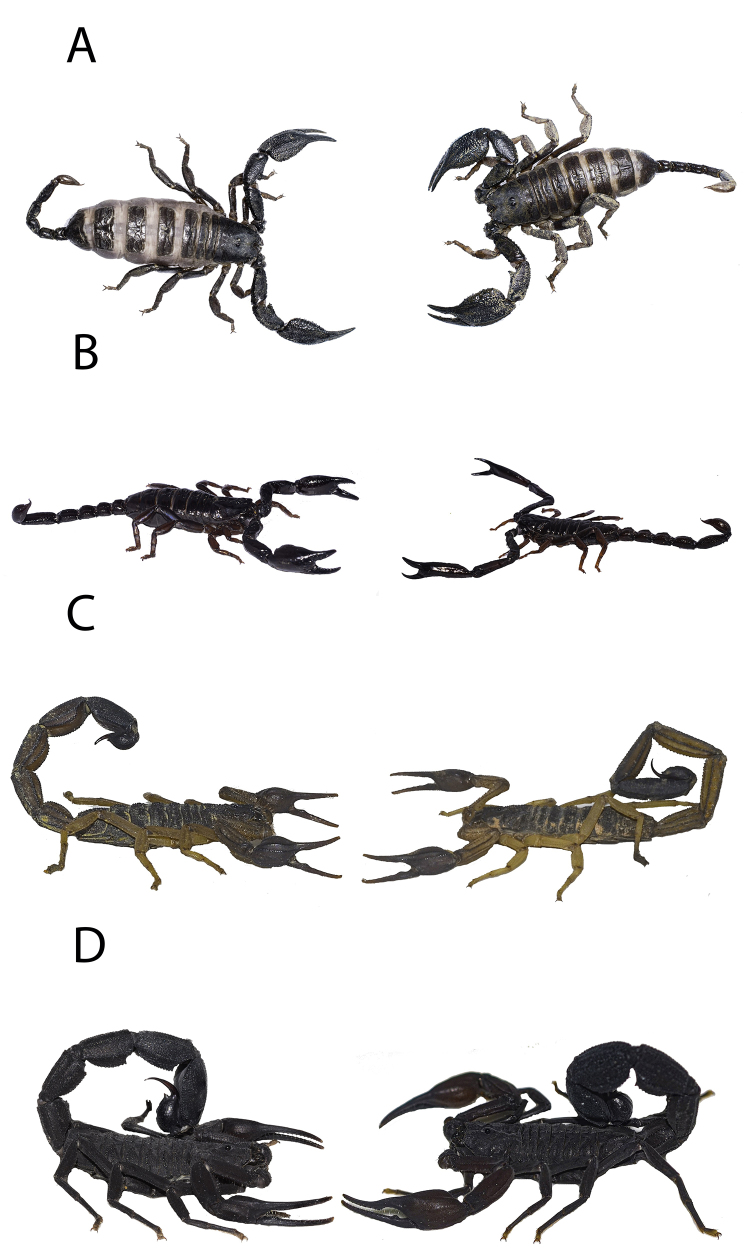
Habitus of the scorpions used on this study. On the left females are presented and on the right, males. **(A)** *Opisthacanthus elatus*, **(B)** *Chactas* sp., **(C)** *Centruroides edwardsii*, and **(D)** *Tityus* sp.

We compared the sting use and feeding efficiency of the selected scorpion species against prey with different morphologies (Figure 1). As a soft prey, we used crickets (*Acheta domesticus* Linnaeus, 1758) and as a hard prey we used cockroaches (*Blaptica dubia* Serville, 1839) (Table 2), as some species of cockroaches are known to have a tough exoskeleton which is able to withstand forces several times higher than their own body weight [28]. To standardize hunger levels, scorpions were fed to satiation two weeks before starting the experiments with *T. molitor* larvae [17]. All experiments were carried outin plastic boxes (12x9x6cm) that were sterilized with 70% alcohol and waterbetween each trial to remove any olfactory remains of the previous experiment. All videos were recorded with a Nikon D7000 camera. We used red LED lights to illuminate the experiments, because these do not affect the scorpions’ behavior [29].

**Table 2.** Length of the prey. All measurements are in mm. Length from head to end of abdomen (mean ± SE).

Species of prey	Prey length
*Acheta domesticus* (*n* = 76)	14.6 ± 0.3
*Blaptica dubia* (*n* = 76)	17.2 ± 0.4

Each prey type was presented randomly to each scorpion, using a complete random block design [30]. In each experiment, we placed the prey at the opposite end of the scorpion’s box (about 6cm away) and recorded the total immobilization time, which was considered as the time interval from the first contact between the scorpion and the prey until it stopped moving. We also recorded whether, and how often, the stinger pierced the prey to paralyzeit. 

Given that relative size might influence prey capture in scorpions, we evaluated the effect of prey:predator size ratio (Additional file 2) on sting use and immobilization time. For scorpions and their prey morphometric data were obtained by photographing individuals with a size standard using a Nikon D7000 digital camera and measuring them with the program ImageJ [31]. We estimated the prey:scorpion size ratio for selected traits, namely the scorpion’s prosoma length and width and chelae length and width (Table 1). Given that size ratio for the morphological traits we selected presented a strong collinearity, we created a new variable (hereafter name Relative Size), by applying a principal component analysis to the prey:scorpion size ratio for the chosen morphological variables and then extracting the first component which explained 93% of the observed variability, as suggested by Zuur et al [32].

### Sting use

We compared sting use on crickets and cockroaches among the different scorpion species. Data were analyzed using a generalized estimating equation with a binomial distribution (GEE-b) [33], given that same individuals were used more than once. Scorpion species, prey type and relative size were used as explanatory variables. Scorpion individual was considered as random variable. In this analysis, we included Stinger Use as a response variable. When not stung, we observed if the scorpion crushed the scorpion. We defined as crush, when the scorpion repeatedly pressed the prey using pedipalps. 

### Immobilization time

To test if immobilization time was different between species, we used log-transformed Immobilization Time as the response variable and we used the Stinger Use, Prey Type and Relative Size as explanatory variables. Relative Size was included as it influences immobilization time in other venomous predators such as spiders [34]. We also looked for potential interactions between Scorpion Species and Stinger Use as well as Scorpion Species and Relative Size. All analyses were made using a GEE with a Gaussian distribution, give the data observed distribution. 

## Results

### Chelae bite force

**Figure 2.**
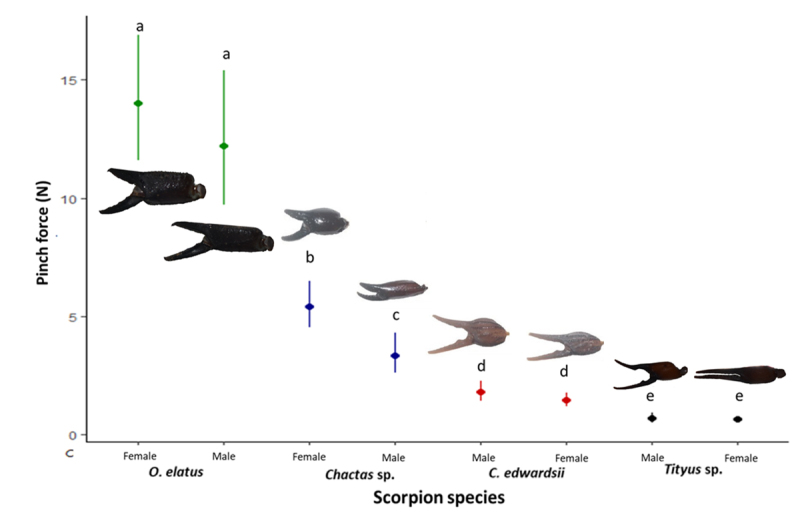
Pinch forces of different species and genders of scorpions. Points are means whereas lines are confidence intervals. Letters indicate significant differences. Parameters were estimated using a linear model. The size of the chelae are not to scale.

We found a marked and significant difference on pinch force between the evaluated species (F_(7,68)_ = 130, p<0.01). Post-hoc comparisons showed the highest force for *O. elatus*, followed by females and males of *Chactas* sp. respectively. Still lower force values were recorded for both sexes of *C. edwardsii*, and the weakest pinch forces were recorded in *Tityus* sp. (Additional file 1) mean forces and confidence intervals are illustrated in Figure 2. 

### Sting use

**Figure 3.**
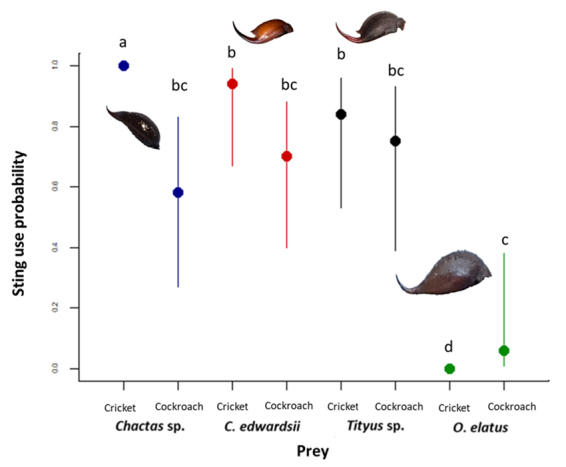
Sting use probability of the different species of scorpions. Points are means and lines are confidence intervals. Letters indicate significant differences. Parameters were estimated using a Generalized Estimated Equation with a binomial distribution. The size of the stingers are not to scale.

We found a significant interaction between scorpion species and prey type (χ^2^_3_ = 4.78 x 10^21^, p<0.01). Some species like *O. elatus* used their sting only against cockroaches while crickets were never stung and their body collapsed several times when crushed by the scorpion’s pedipalps (Figure 3, Additional files 3 and 4). In contrast to the other species, *Chactas* sp. stung both prey, but crickets were always stung (Additional files 5 and 6), while cockroaches were stung less frequently than crickets but in similar proportions to the other scorpion species (Additional file 6). Both buthid species always stung both offered prey types, while holding them with their pedipals (Additional files 7-10). Overall, we did not find a significant effect of relative size (χ^2^_1_ = 2.00, p = 0.12) or sex (χ^2^_1_ = 1.00, p = 0.72) on the stinger use. All videos are also available in a playlist (https://bit.ly/3HOvWRC). 

### Immobilization time

**Figure 4.**
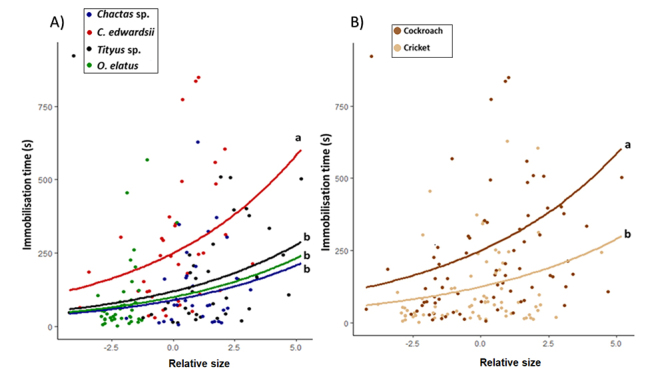
Immobilization time of the different species by: **(A)** scorpion species and **(B)** prey species. Letters indicate significant differences. Parameters were estimated using a generalized estimated equation with Gaussian distribution.

Overall, we found that Immobilization Time significantly increased with the Relative Size (χ^2^_1_ = 4.4, p = 0.035), and we also found significant differences with Scorpion Species (χ^2^_3_ = 34.0, p<0.01) and Prey Type (χ^2^_1_ = 35.4, p<0.01). Post-hoc comparisons showed that immobilization time for *C. edwardsii* was significantly longer than the other species (Figure 4A). When we compared both prey types, we found that the immobilization time for cockroaches was significantly longer when compared to crickets (Figure 4B). Surprisingly, stinger use did not affect the immobilization time (χ^2^_1_ = 0.70, p = 0.40). 

## Discussion

Venom is considered a metabolically expensive product and its usage must be regulated by the scorpions. Several hypotheses have been proposed to explain the optimal use and toxicity in scorpions. According to Evans et al. [26], the use of large pedipalps is often accompanied with small stingers, while the opposite trend is observed in scorpions with slender pedipalps. As a consequence, venom use should be optimized depending on scorpion morphology, being more frequent in scorpions with a low pinch force [5]. 

When we compared the pinch force in all evaluated species, *O. elatus* has the highest pinch force of all evaluated species, followed by *Chactas* sp., where females were stronger males. However, both sexes of *Chactas* sp. had a higher pinch force than both the evaluated buthid species. In *Tityus* sp. we recorded a lower pinch force than in *C. edwardsii*, probably because of the more slender chelae found in the former species. In the particular case of *Chactas* sp., males have slenderer chelae than females, which might explain the higher pinch force of the latter. This agrees with previous studies which suggest that scorpions with robust chelae are stronger than species with slender chelae [4,5,35]. Since we did not correct for overall body size, the between-species comparisons are no indication for relative pinch performance.

We found a different sting use between the studied species. For example, *O. elatus* used their sting occasionally against cockroaches, while crickets were never stung. We hypothesize that pedipalp pinch force in this species is enough to overcome soft and highly mobile prey like crickets, and to overcome most harder prey like cockroaches. This species therefore uses a similar strategy to some predators with massive claws, like some crabs which crush their prey[9]. During prey capture we observed that some structures like head and thorax were repeatedly crushed by *O. elatus*, probably to incapacitate the prey, or to facilitate prey ingestion, similarly to other predators like spiders [36,37]. Cockroaches were more frequently stung than crickets by *O. elatus*, probably as their tough exoskeleton did not collapse under repeated pinches. In both buthid (*Tityus* sp. and *C. edwardsii*) scorpion species, the stinger was used for both prey types, probably because the weaker chelae were not even able to crush soft-bodied prey such as crickets. This may also explain why previous studies found species with slender chelae to more frequently use their stinger against potential predators [21,38]. However, this hypothesis of insufficient force being augmented with stinging needs to be further tested, as there may also be other relevant factors, such as behavioral preferences or the prey’s defensive behavior.

Immobilization time was affected by prey: predator size ratio. This is an expected result given that larger prey are usually harder to subdue than smaller ones [39]. Similarly, large prey might require more venom to be subdued because of their size and mass. This may be why, even when stung, very large prey were hard to paralyze. However, this would suggest that scorpions do not release enough venom on the first sting when estimating prey size, but instead measure venom by applying multiple consecutive stings until the prey stops moving [40]. Such behavior may be a function of the size of the prey, as it has been demonstrated in *Hadrurus spadix* Stahnke, 1940, which also shows a positive relationship between sting use and prey size [40]. However, prey size and prey activity may both influence venom administration [41]. Although we expected scorpions with a similar morphology to have similar immobilization times, this was not the case, since all scorpions had similar immobilization times against offered prey except for *C. edwarsii*. Interestingly, scorpions with contrasting morphologies such as *Tityus* sp. and *O. elatus* displayed similar performance against offered prey, independent of whether these were hard or soft-bodied. This suggests that by using venom and/or chelae, incapacitation performance was similar between these species. A similar trend has been observed in some snakes where constriction might be equally or more effective than the use of toxins when subduing prey, underlining the importance of mechanical strategies during prey incapacitation [42,43].

Although not significantly different, we observed that immobilization time was shorter for *Chactas* sp. than for the other species, suggesting that it was slightly more efficient than both aforementioned species. This might be due to *Chactas* sp. using both strong chelae and stinging during prey capture. However, since *Chactas* sp. was also the smallest species we studied, this difference may also be a result of scaling effects. Although their morphologies and prey capture strategies are similar, *C. edwardsii* and *Tityus* sp. were not similarly effective in prey incapacitation, and the former species was less efficient than the latter during prey capture. We hypothesize that the differences recorded in predatory efficiency between *C. edwardsii* and *Tityus* sp. might be explained by a difference in the toxicity of the venom. Although a high insecticidal toxicity has been reported for several *Tityus* species [44,45], the effect of *C. edwardsii* venoms against potential insect prey is reported to be variable, with crickets being more susceptible, while the cockroaches and the mealworms are more resistant [46].

When comparing capture efficiency against the offered prey species, we observed that cockroaches were harder to immobilize than crickets, which may be due to the former having a tougher body with a more difficult to penetrate exoskeleton. Cockroaches also have been reported to be more resistant against some toxins like those of some spider species [47] and other scorpions like *C. edwardsii*. Crickets have been reported to be more susceptible to scorpion venom [46], and are also soft-bodied, making them more susceptible to being crushed.

Although using a limited number of prey and predator species, this is to our knowledge the first study to compare the role of chelae and stinging in prey capture in scorpion species with contrasting morphologies.

## Conclusion

We found that scorpions with robust chelae and slender metasoma such as *O. elatus*, not only have a high pinch force, but also a reduced sting use, suggesting that prey crushing is the main prey incapacitation strategy for this species, even when facing hard-bodied prey. The two buthid (*Tityus* sp. and *C. edwardsii*) species used the stinger more frequently to incapacitate their prey. Interestingly, we identified the scorpion *Chactas* sp. as using a mixed strategy, with high pinch force and sting use that allowed them to overcome quickly both prey types offered (Additional files 5 and 6). In the case of buthid scorpions, although both species used the same strategy (forces and sting use), we found differences in immobilization time, which might be explained by a difference in the toxicity of the venom to insects. Although our study shows a trade-off between pinch force and sting use in some species and mixed strategy in others, further studies should explore if the trends observed for the species on this study are applicable for other scorpions with similar morphologies, and could include other effects of prey morphology and behavior such as dangerous or highly active prey.

## Supplementary material

The following online material is available for this article:

Additional file 1. Force measurements (in Newtons) in different species of scorpions.

Additional file 2. Prey:predator size ratio (mean ± SD) for the different morphological measurements.

Additional file 3. Video showing *Opisthacanthus elatus* feeding on a cockroach (*Blaptica dubia*).

Additional file 4. Video showing *Opisthacanthus elatus* feeding on a cricket (*Acheta domesticus*).

Additional file 5. Video showing *Chactas* sp. feeding on a cockroach (*Blaptica dubia*).

Additional file 6. Video showing *Chactas* sp. feeding on a cricket (*Acheta domesticus*).

Additional file 7. Video showing *Tityus* sp. feeding on a cockroach (*Blaptica dubia*).

Additional file 8. Video showing *Tityus* sp. feeding on a cricket (*Acheta domesticus*).

Additional file 9. Video showing *Centruroides edwardsii* feeding on a cricket (*Acheta domesticus*).

Additional file 10. Video showing *Centruroides edwardsii* feeding on a cockroach (*Blaptica dubia*).
