# Supplementary material for: Pinching or stinging? Comparing prey capture among scorpions with contrasting morphologies
Source: J Venom Anim Toxins Incl Trop Dis. 2022 Apr 1;28:e20210037. doi: 10.1590/1678-9199-JVATITD-2021-0037 (PMC8985449; doi:10.1590/1678-9199-JVATITD-2021-0037)
Supplement: Additional file 1. [file 1678-9199-jvatitd-28-e20210037-s1.pdf]

J Venom Anim Toxins incl Trop Dis
https://doi.org/10.1590/1678-9199-JVATITD-2021-0037

Supplementary Material to "Pinching or stinging? Comparing prey capture among scorpions with contrasting morphologies"

Additional file 1 - Forces of the left and right chelae of scorpion species. Measurements in Newtons (mean ± SE).

Species	Sex	Chelae	
		**Left**	**Right**
Centruroides edwardsii	Female	0.99 ± 0.60	0.92 ± 0.53
	Male	0.67 ± 0.46	0.69 ± 0.45
Chactas sp.	Female	4.23 ± 1.17	3.89 ± 1.23
	Male	2.64 ± 1.10	2.36 ±1.07
Opisthacanthus elatus	Female	11.07 ± 3.88	10.64 ± 3.59
	Male	9.71 ± 3.63	9.49 ± 3.16
Tityus sp.	Female	0.37 ± 0.19	0.39 ± 0.20
	Male	0.39 ± 0.23	0.38 ± 0.28
