# Supplementary material for: Pinching or stinging? Comparing prey capture among scorpions with contrasting morphologies
Source: J Venom Anim Toxins Incl Trop Dis. 2022 Apr 1;28:e20210037. doi: 10.1590/1678-9199-JVATITD-2021-0037 (PMC8985449; doi:10.1590/1678-9199-JVATITD-2021-0037)
Supplement: Additional file 2. [file 1678-9199-jvatitd-28-e20210037-s2.pdf]

J Venom Anim Toxins incl Trop Dis
https://doi.org/10.1590/1678-9199-JVATITD-2021-0037

Supplementary Material to "Pinching or stinging? Comparing prey capture among scorpions with contrasting morphologies"

Additional file 2 - Prey:predator size ratio (mean ± SD) for the different morphological measurements scorpions.

	Size ratio			
**Species**	**Prosoma length**	**Prosoma width**	**Chela length**	**Chela width**
Centruroides edwardsii	1.91 ±0.35	2.35 ± 0.39	1.16 ± 0.24	4.10 ± 1.43
Chactas sp.	2.28 ± 0.39	2.74 ± 0.52	1.21 ± 0.28	4.56 ± 1.13
Opisthacanthus elatus	1.72 ± 0.30	1.78 ± 0.9	0.90 ± 0.21	2.27 ± 0.42
Tityus sp.	2.35 ± 0.86	2.76 ± 1.00	1.27 ± 0.49	5.53 ± 2.09
