# Supplementary material for: Pinching or stinging? Comparing prey capture among scorpions with contrasting morphologies
Source: J Venom Anim Toxins Incl Trop Dis. 2022 Apr 1;28:e20210037. doi: 10.1590/1678-9199-JVATITD-2021-0037 (PMC8985449; doi:10.1590/1678-9199-JVATITD-2021-0037)
Supplement: Additional file 9. [file 1678-9199-jvatitd-28-e20210037-s9.zip › 1678-9199-jvatitd-28-e20210037-s9.pdf]

J Venom Anim Toxins incl Trop Dis
https://doi.org/10.1590/1678-9199-JVATITD-2021-0037

Supplementary Material to “Pinching or stinging? Comparing prey capture among scorpions with contrasting morphologies”

Additional file 9 - Video showing *Centruroides edwardsii* feeding on a cricket (*Acheta domesticus*).
